# Advanced Omics Techniques for Understanding Cochlear Genome, Epigenome, and Transcriptome in Health and Disease

**DOI:** 10.3390/biom13101534

**Published:** 2023-10-17

**Authors:** Annamaria Tisi, Sakthimala Palaniappan, Mauro Maccarrone

**Affiliations:** 1 Department of Biotechnological and Applied Clinical Sciences, University of L’Aquila, 67100 L’Aquila, Italy; sakthimala.palaniappan@graduate.univaq.it; 2 Laboratory of Lipid Neurochemistry, European Center for Brain Research (CERC), Santa Lucia Foundation IRCCS, 00143 Rome, Italy

**Keywords:** omics, cochlea, single-cell omics, spatial omics, epigenomics, transcriptomics, genomics, organ of Corti

## Abstract

Advanced genomics, transcriptomics, and epigenomics techniques are providing unprecedented insights into the understanding of the molecular underpinnings of the central nervous system, including the neuro-sensory cochlea of the inner ear. Here, we report for the first time a comprehensive and updated overview of the most advanced omics techniques for the study of nucleic acids and their applications in cochlear research. We describe the available in vitro and in vivo models for hearing research and the principles of genomics, transcriptomics, and epigenomics, alongside their most advanced technologies (like single-cell omics and spatial omics), which allow for the investigation of the molecular events that occur at a single-cell resolution while retaining the spatial information.

## 1. Introduction

According to the World Health Organization (WHO), 432 million adults and 32 million children are affected by disabling hearing loss, and it is estimated that this number will increase to 700 million by 2050 [1]. In particular, sensorineural hearing loss (SNHL) is characterized by the deterioration of the neuro-sensory structure of the inner ear—the cochlea—and leads to irreversible hearing loss that affects communication, speech, and cognition, with a clear impact on the quality of life and severe socio-economic consequences. SNHL can be caused by either congenital or acquired factors (noise exposure, ototoxic drugs, ageing, strial or metabolic dysfunctions) [2]. The severity of the aetiology can range from synaptic disconnectivity of the sensory epithelium [3]—composed of inner (IHCs)/outer hair cells (OHCs) and supporting cells (SCs)—to critical cases of the loss of hair cells (HCs). The latter process is often followed by the degeneration of the downstream spiral ganglion neurons (SGNs) [4], whose axons form the auditory nerve. Although cochlear implants and hearing aids exhibit some beneficial outcomes in deaf patients, they cannot entirely replace the cochlea’s functionality [5]. Thus, management-based approaches must give way to disease-modifying interventions. This strategy needs a more thorough understanding of the molecular events that could eventually become novel therapeutic targets and/or diagnostic biomarkers of SNHL, to be exploited also in cochlear regeneration strategies. Thanks to the technological advancements in the field of molecular biology, recent progress has been made in identifying and characterizing novel genes involved in hearing loss [6], as well as new molecular mechanisms of cochlear development [7], degeneration, and regeneration [8]. In this review, we aim to present cutting-edge molecular methodologies that have been used to investigate the genome, epigenome, and transcriptome in cochlear research, as well as methods that could be employed in the future to expand our understanding in the field, such as the latest sophisticated single-cell and spatial genomics, transcriptomics, and epigenomics.

## 2. Experimental Models in Inner Ear Research

**Figure 1:**
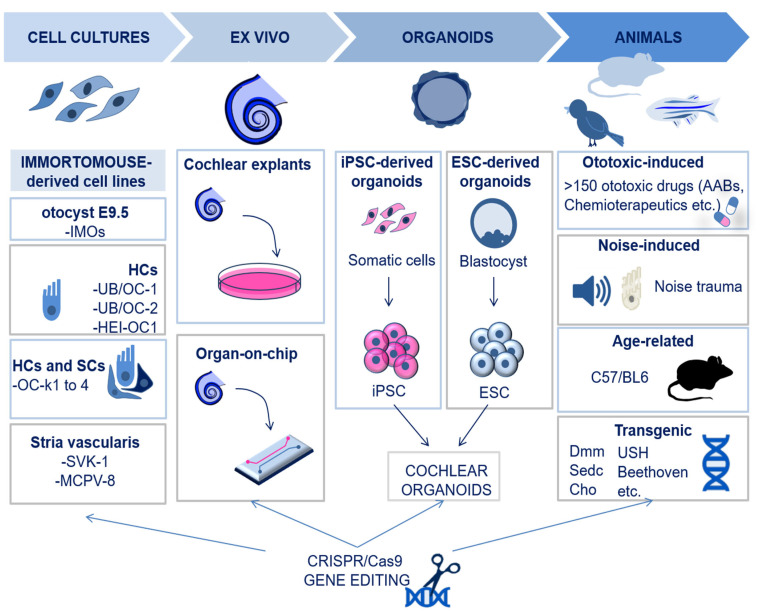
Schematic illustration of the available experimental models for cochlear research. The available models for cochlear research include cell lines of otocyst, HCs, organ of Corti, and stria vascularis. Explants of cochlear tissues may also be used, more recently via microfluidic chambers for organ-on-chip cultures. Cochlear organoids are an additional in vitro possibility and can be derived from induced pluripotent stem cells (iPSCs) or from embryonic stem cells (ESCs). Animal models can be generated by exposure to ototoxic drugs or by noise trauma; also, age-related and transgenic models of hearing loss have been developed. Finally, all the models may be subjected to CRISPR/Cas9 to achieve targeted gene editing. Abbreviations: IMO; Immortomouse; HC; hair cell, SC; supporting cell, iPSC; induced pluripotent stem cell, ESC; embryonic stem cell, Dmm; disproportionate micromelia; sedc, spondyloepiphyseal dysplasia congenital; USH: Usher; Cho: chondrodysplasia.

Modelling inner ear disorders is important to understand the molecular basis of hearing, as well as the mechanisms of deafness in humans. Currently, it is only possible to study human inner ear disorders in cadavers [9] since sampling tissues from alive subjects would cause irreversible damage to the intricate inner ear structures. Hence, this is possible only in cases of inner ear tumors [10,11]. Moreover, non-invasive techniques, such as magnetic resonance imaging (MRI) and computerized tomography (CT), cannot lead to a detailed understanding of the inner ear pathogenesis [12]. Therefore, most of the models for studies on the cochlea are based on cell cultures from animals or on animal models. Figure 1 summarizes the experimental models that are currently available and used in cochlear research.

### 2.1. In Vitro and Ex Vivo Models: Cochlear Cell Lines, Organotypic Cultures, and Organoids

The establishment of cochlear cell cultures has been challenging for a long time due to the paucity of the tissue and the poor accessibility of the inner ear. With the advent of the Immortomouse™, a transgenic mouse model carrying the temperature-sensitive tsA58 variant of the SV40 T-antigen, it became possible to develop immortalized cell lines from the inner ear [13]. Therefore, to date, most of the available cell lines are derived from Immunomortomouse^TM^, including the Immunomortomouse^TM^ otocysts E9.5 (IMOs), HC cultures (like UB/OC-1, UB/OC-2, HEI-OC1), and cells of the organ of Corti, which include either HCs or SCs (e.g., OC-k1to 4). The only human inner ear cell line developed so far is the immortalized endolymphatic sac (ES) cell line [14], while none have been developed yet for the human organ of Corti. In addition to cell cultures, cochlear explants (or organotypic cultures) are another efficient experimental tool to identify and characterize molecular and genetic pathways which play a role in the specification and patterning of cells in their natural environment [15]. A recent improvement in organotypic cochlear models is based on the use of microfluidic chambers for organ-on-chip culture, which allows us to reproduce a more controlled microenvironment [16]. However, cochlear explants require the use of a large number of animals, and there are technical issues with the isolation of the intact tissue to be cultured. Inner ear organoids, derived from induced pluripotent stem cells (iPSCs) or embryonic stem cells (ESCs), represent a relevant alternative to recapitulate the physiological dynamics of the cochlea in terms of cell type yield and functionality, particularly for HCs. However, it is only claimed that a small portion of the organoid cultures contain functional sensory HCs, and the reason why this happens is not yet fully understood [17]. It should be stressed that the optimization of inner ear organoid culture could lead to better drug screening programs and disease modelling opportunities.

### 2.2. In Vivo Models

Animal models used in hearing research are based on multiple species (rats, guinea pigs, mice, chinchillas, gerbils, birds, and zebrafish) that differ in the physiological and anatomical characteristics of the auditory system and offer different view angles to study the inner ear [18]. These animals include genetic models of spontaneous or inherited hearing loss, carrying mutations for specific genes associated with hearing impairment in humans. For instance, mutations in the genes encoding for collagen and non-collagen proteins (like α-tectorin) that are important for the structure of the basilar membrane (BM) and the tectorial membrane (TM) in the organ of Corti cause SNHL. These mutations successfully recapitulate cochlear degeneration in mice; for instance, disproportionate micromelia (Dmm) mice, spondyloepiphyseal dysplasia congenita (sedc) mice, Col2a1G574S mice, Col9a1 KO mice, and chondrodysplasia (Cho) mice all carry mutations in collagen genes; Tecta^∆ENT/∆ENT^ and Tecta^Y1870C/Y1870C^ both carry a mutation in the gene encoding for α-tectorin, *Tecta* [19,20]. Also, mutations in genes encoding for myosin and other proteins important for HC function and mechanotransduction, as well as genes encoding for endolymph proteins, are associated with SNHL, and multiple genetic models have been successfully developed: Beethoven mice (mutations in TMC1), Usher syndrome models, and Lcc and Ysb mice [21,22]. In addition to genetic predisposition, SNHL may also be caused by external noxious events. As of now, more than 150 established ototoxic substances have been identified, the most widely used being aminoglycoside antibiotics (AABs), loop diuretics, and antitumor medications [23]. The administration of these substances to animals recapitulates cochlear degenerative events observed in humans and is helpful in the study of degenerative mechanisms and neuroprotective strategies [18]. Additionally, exposure to loud sounds is an important risk factor for noise-induced hearing loss (NIHL); hence, noise trauma can be used to successfully reproduce NIHL in animals as well. Of note, rodents are more susceptible to noise trauma compared to non-human primates, suggesting different degenerative mechanisms with important implications from a translational point of view [24].

### 2.3. New Models Created by CRISPR/Cas9 Technology

The CRISPR/Cas9 technology has been developed following years of research on adaptive immunity in prokaryotes [25,26,27]. Engineering of this machinery has allowed the performance of gene editing in terms of base and prime editing, as well as the knock-out/ knock-in of genes. Therefore, the CRISPR/Cas9 technology represents a powerful tool for basic molecular studies in hearing research and a promising strategy for therapeutic approaches to SNHL [28,29,30]. In particular, CRISPR/Cas9 has been successfully applied to the creation of new in vitro and in vivo models of cochlear diseases [31]. For instance, this technology has allowed the study of genes associated with ototoxicity via the knock-out of Lim-domain only 4 (LMO4), for cisplatin, and HtrA Serine Peptidase 2 (htra2), for aminoglycosides, in vitro and in vivo models, respectively [32,33]. It has also allowed the study of inherited hearing loss genes, such as MYO7A, CIB2, and CDH23, for Usher syndrome [31]. Moreover, zebrafish models to study genes involved in the development of the auditory system, such as POU4F3, have recently been developed based on CRISPR/Cas9 [34].

## 3. Omics Techniques

### 3.1. Introduction to Omics: Principles and Advancements

The term omics refers to a rapidly evolving and expanding group of techniques aimed at investigating pools of biological molecules of an organism, including nucleic acids, proteins, and metabolites [35]. Hence, the main branches of omics techniques are known as genomics, epigenomics, transcriptomics, proteomics, and metabolomics. The most advanced omics techniques include single-cell omics and spatial omics, which allow the investigation of the molecular events occurring at a single-cell resolution and the retention of spatial information [36,37]. There are also other advanced and upcoming sequencing-based omics, such as epitranscriptomics, epiproteomics, and interactomics (DNA–RNA, RNA–RNA, RNA–protein, protein–protein, protein–metabolite), which give detailed information on the complex interactions and dynamics of regulation in a biological system [35]. The number of omics studies in cochlear research is relatively low compared to other sensory systems, since the sampling of the cochlear tissues has only recently advanced, and some techniques are incompatible with the small sample quantity obtained [38]. Nonetheless, the studies performed so far have significantly advanced the knowledge of cochlear physio-pathology.

In this review, we focus on the omics techniques that target nucleic acids, which are genomics, transcriptomics, and epigenomics in bulk, single, and spatial resolution. We also provide evidence that the availability of these techniques has been transformative in unraveling novel molecular signatures in hearing research, advancing our understanding of the treatment of cochlear degenerative diseases.

### 3.2. Principles of Single-Cell Omics

The term single-cell omics refers to the process of profiling the genome, transcriptome, epigenome, proteome, and metabolome in individual cells. As a consequence, single-cell techniques were shown to be useful in several biological fields, including cancer [39], developmental biology [40], stem cell research [41], neuroscience [42], and hearing [8].

The first step of all these technologies is the isolation of individual cells and the setting up of libraries. Multiple methodologies have been designed to isolate single cells from pooled cell populations/tissues through a variety of techniques [43] that span from the most straightforward—using pipettes and cell isolation by dilution—to the more sophisticated—using advanced microfluidic technologies [44]. The latter include hydrodynamic trapping, droplet-based isolation, valve-based isolation, microwell-based isolation and dielectrophoresis trapping [45], as well as magnetic-activated cell sorting (MACS), flow-activated cell sorting (FACS) [43], laser capture microdissection (LCM)—which also preserves spatial context—and nanowell-based cell sorting [46]. Details of the isolation methods for abundant or rare cells have been described by Wang and Navin [47].

After the isolation of single cells, the genome, the epigenome and the transcriptome can be profiled [8,48]. Notably, single-cell multi-omics approaches have recently been developed to investigate the molecular events that occur in individual cells under physiological or pathological conditions in a wider overview, at once. An example of this cutting-edge methodology is single-cell triple-omics sequencing (scTrio-seq), which simultaneously gathers data from the genome, DNA methylome, and transcriptome of a single cell [49].

### 3.3. Spatial Omics

The study of omics at a single-cell resolution has been transformative in the identification of novel biomarkers and molecular regulators of tissues, yet single-cell omics cannot deliver information on the tissue or sub-cellular localization of the isolated cells. For this reason, spatial omics have been developed with the aim of identifying molecular events while maintaining the spatial information. Multiple spatial omics approaches exist, and they vary depending on the biomolecules of interest. In the cochlea, spatial omics are of particular relevance due to its complex anatomical architecture. Indeed, the cochlea exhibits a tonotopic organization from its base (high-frequency perception) to the apex (low-frequency perception), which requires appropriate cellular structures and expression patterns [50]. Moreover, different cell types are also present from the medial (i.e., greater epithelial ridge (GER), IHCs, and their associated SCs) to the lateral (i.e., Deiters’ cells, pillar cells, and OHCs) compartment of the cochlea [50]. Hence, entering the spatial era can deepen our understanding of the cellular organization and interplay in regions of interest.

## 4. The Role of Bioinformatics in Analyzing Omics Data

In order to fill the knowledge gap between omics data acquisition and interpretation, bioinformatics is a critical field. Numerous computational techniques have been developed to this end, including machine learning, deep learning, data mining and statistical and metaheuristic approaches, to analyze, process, interpret, and integrate omics data for both single omics and integrative multi-omics [51,52,53,54,55]. Machine learning and deep learning are frequently used in the research community for decoding and analyzing data, predicting disease occurrence and recurrence, calculating survival rates, and finding potential biomarkers [51]. Deep learning models are a sub-set of machine learning tools of high utility since they are automated and analyze large high-dimensional data sets. Deep learning is primarily based on stratified artificial neural networks, providing diverse interpretations based on the fed data. The primary neural networks in deep learning include recursive neural networks (RvNNs), recurrent neural networks (RNNs), and convolutional neural networks (CNNs) [56]. Given the recent rapid advancements in omics and the accumulation of high-throughput omics data, future efforts should be aimed towards improving current machine learning and deep learning models for multi-omic data analysis. In this context, graph neural networks (GNNs) have gained attention in recent years [57]: the spatial relations within and between cells can be better represented with graph models, and graph-based artificial intelligence appears to hold promise, especially with regard to the most advanced omics (i.e, spatial omics). In this context, it should be mentioned that two relevant techniques have recently been developed to analyze spatial transcriptomics data: SPAcI [58] and SiGra [59]. They both have several technical advantages over existing methods, such as an improvement in accuracy, enhancing noisy gene expression data sets, and an increased ability to adapt [57,58].

## 5. Genomics

### 5.1. Principles of Sequencing

**Figure 2:**
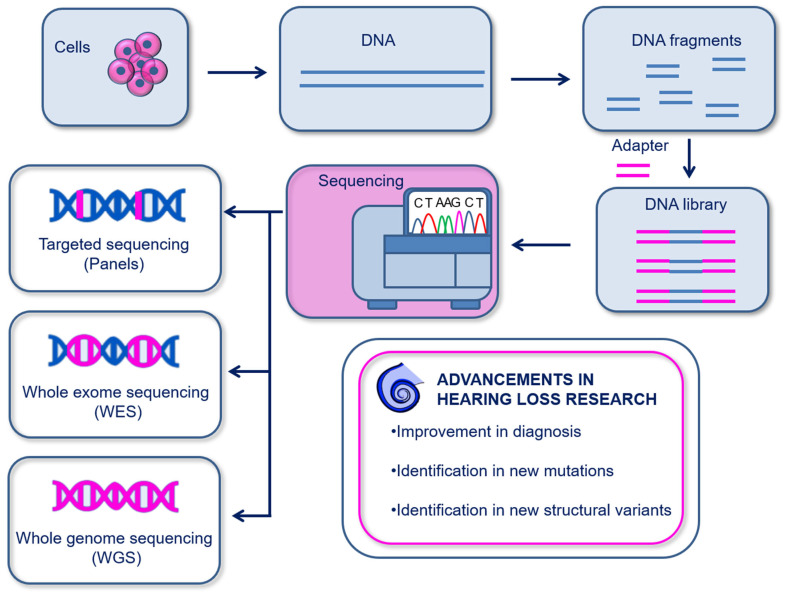
Schematic illustration of genomics. DNA is isolated from cells or tissues and is fragmented in order to create DNA libraries using DNA adapters. Sequencing can then be performed on targeted sequences (panels), on the whole exosome (WES), or on the whole genome (WGS). Genomics has provided important advancements in the diagnosis and discovery of genetic hearing loss.

Genomics investigates somatic and germ-line inter-individual variations in the genome. The currently most used genomics are based on sequencing for the determination of the nucleic acid sequence. Genomics has been used to identify several genetic disorders and to disclose novel alleles in multiple inherited human diseases [35], including hearing loss [60,61,62,63,64,65]. The first sequencing method, known as the chain-termination method, was first developed by Sanger in 1977 and was based on the capillary electrophoresis of fragmented DNA bound to a single-stranded DNA template. The main drawback of Sanger sequencing is the ability to sequence only a low amount of DNA at a time [66]. To date, more advanced sequencing technologies have been developed and allow massive, faster, and more precise sequencing of nucleic acids. These are next generation sequencing (NGS)—more widely used—and third generation sequencing (TGS). The primary difference between these two techniques is the DNA read length. In NGS, the DNA is cleaved in small fragments (150–1000 bp), then amplified and sequenced; instead, TGS uses single-molecule sequencing without the need for prior amplification and reads long DNA sequences at a time. Moreover, it is possible to sequence different lengths of the genome depending on the experimental purpose: targeted genes (targeted panel sequencing), whole-exome sequencing (WES) [62,63] or whole-genome sequencing (WGS) [64,65] (Figure 2). In Table 1, we summarize details of the existing advanced sequencing methods and how they work.

### 5.2. Single-Cell and Spatial Genomics

Single-cell DNA sequencing (scDNAseq) allows the DNA profiling of individual cells [72] and is generally based on NGS. The whole genome of single cells can be primarily amplified using three methods: (i) the degenerate oligonucleotide-primed PCR (DOP-PCR), (ii) the multiple displacement amplification (MDA), and (iii) the multiple annealing and looping-based amplification cycles (MALBAC) [73]. Recently, a single-cell WGS method based on TGS was also developed in order to sequence long reads; this is known as “single-molecule real-time sequencing of long fragments amplified through transposon insertion” (SMOOTH-seq) [73]. SMOOTH-seq has greatly improved the identification of structural variants (SVs) and extra-chromosomal DNA compared to NGS [74]. Also, spatial genomics has recently been developed, but it is mostly used in cancer research to dissect the cellular genome heterogeneity of tumoral cells [75].

### 5.3. Genomic Studies Have Delivered Unprecedented Knowledge on the Genetic Background and Early Diagnosis of Inherited Hearing Loss

Genetic hearing loss affects any part of the auditory system and accounts for ~50% of the deaf population. It can be either non-syndromic (70%) [76,77] or syndromic (30%) [78]. The large heterogeneity of genes involved in deafness makes it difficult to study and diagnose it [62]. However, thanks to the advancements in genomics, to date several variants have been identified in genes associated with hearing loss. For instance, the combination of WES, qPCR, and TGS was able to unravel for the first-time novel SVs of centrosomal protein 78 (CEP78), a key gene responsible for hearing loss associated with cone–rod dystrophy (CRDHL) [65]. The applications of advanced genomics have also revealed new variants that have recently been outlined in important hearing loss-related genes, namely, myosin 15 A (MYO15A), otoferlin (OTOF), radixin (RDX) [79], TATA-box-binding protein-associated factor 1 (TAF1) [80], atonal BHLH transcription factor 1 (ATOH1) [81], and centrosomal protein 78 (CEP78) [65]. The discovery of novel variants represents a fundamental step forward in the understanding of the molecular basis of cochlear diseases, and indeed, it has improved the diagnosis of genetic hearing loss, as well as the prediction of its severity and prognosis. So far, several studies have benefitted from genome sequencing (via either WES or WGS) for the early detection of hearing loss [82,83,84]. For instance, a recent study has shown that the combination of conventional hearing screening and extended genetic sequencing improves the early diagnosis of inherited hearing loss in newborns, with important implications for their clinical management [85]. Yet, genetic conductivity and SNHL are a common occurrence among newborns, whose diagnosis is often missed due to the lack of proper genetic screening at birth.

Overall, the use of genomics has been useful in revealing novel gene variants linked to hearing loss, and thus, it represents a potent diagnostic tool for the genetic screening of inherited deafness.

## 6. Transcriptomics

**Figure 3:**
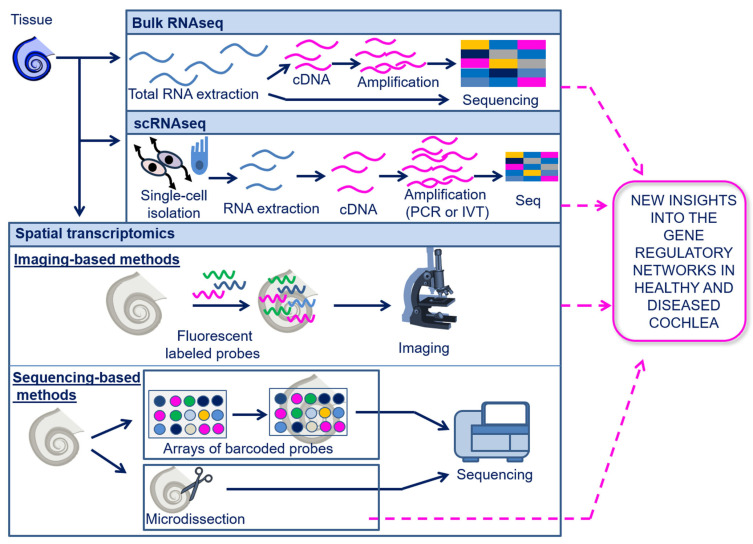
Schematic illustration of transcriptomics. Transcriptomics can be performed at a bulk, single-cell, or spatial resolution. In bulk RNAseq, total RNA is extracted from the tissue and can be directly sequenced or converted into cDNA and then sequenced. In scRNA-seq, the sequencing of the RNA is limited to single cells that are isolated from the tissue and analyzed individually. In spatial transcriptomics, the transcriptome may be analyzed with imaging-based methods, using fluorescent labeled probes which bind to the RNA on tissue slides, followed by microscopic analysis; spatial transcriptomics may also be performed through sequencing-based methods using arrays of barcoded probes or microdissection of target tissue areas, both followed by sequencing. Array-based spatial transcriptomics have not yet been applied in cochlear research. The other transcriptomics techniques have provided important new insights into the gene regulatory networks of the cochlea, under both physiological and pathological conditions. Abbreviations: Bulk RNA seq, Bulk RNA sequencing; scRNA-seq, single-cell RNA sequencing.

Transcriptomics enable the analysis of gene expression at the RNA level, including messenger RNAs (mRNAs), transfer RNAs (tRNAs), ribosomal RNAs (rRNAs), and other non-coding RNAs (ncRNAs) (e.g., microRNAs (miRNAs), long-non-coding RNAs (lncRNAs), and circular RNAs (circRNAs)) [35,86]. As for genomics, the currently most used transcriptomics technologies are based on sequencing (described above in Section 5.1); however, transcriptome profiling is more challenging compared to genome sequencing due to the highly dynamic nature of the transcriptome in biological processes. The sequencing of the entire transcriptome in a tissue or cell population is known as bulk-RNAseq and can be performed either with direct RNA sequencing (dRNA-seq) or with cDNA sequencing [87]. Additionally, the transcriptome can also be investigated at a single-cell and spatial resolution, as detailed in the following paragraphs. A schematic overview of the transcriptomics techniques is shown in Figure 3.

### 6.1. Single-Cell Transcriptomics

Single-cell RNA sequencing (scRNA-seq) allows the RNA profiling of individual cells. Notably, most of the single-cell omics developed so far have been focused on the transcriptome [47,48,88]. The currently available high-throughput platforms for scRNAseq require retro-transcription of the RNA into cDNA. The cDNA is then amplified for the preparation of sequencing libraries. The amplification methods may be based on PCR or in vitro transcription (IVT) and are followed by sequencing through different platforms [88,89]. Among the existing platforms for scRNA-seq, it is noteworthy to mention SMART/SMARTseq2, CEL-seq/CEL-seq2, 10X Genomics, Drop-seq, inDrop, and seq-well [48,89]. To date, the most used commercial platform is 10X Genomics Chromium, which is a droplet-based scRNA-seq technology (10X Genomics, Pleasanton, CA, USA). An important improvement has been made in scRNA-seq which allows for the sequencing of dozens of thousands of cells individually, thanks to the development of microfluidics-based (e.g., Drop-seq [90], inDrop [43,91], and nanowell-based (such as the seq-well) methods [92]. Recently, single-nuclei RNA sequencing (snRNA-seq) has also been developed to improve the quality of scRNA-seq by mitigating the expression changes that can be induced by enzymatic cellular dissociation methods; snRNA-seq is also used to study gene expression under particular conditions, such as those where it is difficult to recover intact cells [93,94]. However, it is important to outline that snRNA-seq does not include cytoplasmatic RNAs; hence, it could hide important information needed to fully characterize the cell transcriptome. Although scRNA-seq has advanced our understanding of cell heterogeneity, it requires cell lysis, which hampers the follow-up molecular analysis on the same cell. To date, it has been difficult to track changes in a cell’s ground-state characteristics to its downstream signaling. Thanks to the advent of Live-cell omics, a state-of-the-art technique coupling Fluidforce microscopy (Fluid FM)-based cytoplasmic biopsy and low-input RNA-seq (as low as 1 picogram) workflow, it is now possible to profile the transcriptome, as well as the molecular/functional changes of the same cell at different time points, while preserving cell viability. This methodology is known as live-seq and can enhance the knowledge on cell dynamics and regulation [95].

### 6.2. Spatial Transcriptomics

The spatial omics for transcriptome studies are broadly divided into imaging-based technologies (where RNA is detected using fluorophores on intact tissues and then detected by microscopy) and sequencing-based technologies (based on RNA capturing from the tissue, followed by NGS) [75,96,97,98].

#### 6.2.1. Imaging-Based Technologies

One of the primary imaging-based methodologies for spatial transcriptomics is fluorescent in situ hybridization (FISH), which includes SeqFISH [99], SeqFISH+, and MERFISH [100]. The principle of FISH is the hybridization of fluorescent gene-specific probes to nucleic acids on a tissue section directly, which is then analyzed through microscopy. A recent improvement in FISH is the enhanced electric FISH (EEL-FISH). In EEL-FISH, tissue mRNAs are electrophoretically transferred onto glass coverslips and are then hybridized. In this way, it is possible to accelerate data collection due to a reduced need for acquired images compared to the other FISH techniques [101]. Indeed, since the acquisition analysis is performed on coverslips and not on the tissue directly, the time of acquisition is shortened because it is not necessary to image the z axis of the tissue section. Notably, today it is also possible to visualize 3D gene expression in a tissue, thanks to the development of the expansion-assisted iterative fluorescence in situ hybridization (EASI-FISH) methodology [98,102]. Another imaging-based approach for spatial transcriptomics is in situ sequencing (ISS), where nucleic acids are first amplified (preserving spatial localization through rolling circle amplification). Differently from FISH, ISS does not use gene-specific probes; instead, it employs probes for 1–2 nucleotides at a time linked to distinct fluorophores and visualization through microscopy, which leads to the identification of the transcripts [97,98,103].

#### 6.2.2. Sequencing-Based Technologies

Sequencing-based technologies allow the sequencing of the RNA from a tissue section through NGS. The mRNA is first captured in the tissue and then retrotranscribed to cDNA followed by calculating gene-specific sequences using NGS. Importantly, the spatial information is also retained because of the recording of the specific location where the RNA is captured. Sequencing-based techniques include “microdissection-based” and “array-based” methodologies [98]. Microdissection-based methods allow sequencing (via different platforms) of a specific portion of a tissue that is microdissected with dedicated instruments. Hence, the main limitation is the low spatial resolution. Microdissection-based technologies include laser capture microdissection combined with NGS (LCM-NGS) [104], Tomo-seq [105], Geo-seq [106], GeoMx DSP [107,108], and STRP-seq [109].

Differently, array-based technologies employ arrays with spatially barcoded probes: the RNAs are retro-transcribed in cDNAs that are then sequenced. In this case, the spatial resolution depends on the area of the barcode and is therefore higher compared to microdissection-based methods [98]. To date, Visium by 10X Genomics has achieved a spatial resolution of 2 μm [110,111], while the Stereo-seq is capable of achieving an even a lower resolution, up to 0.5 μm [112]. Other notable techniques for spatial transcriptomic analysis also include slide-seq and slide-seqV2 [75,113,114] and the deterministic barcoding in tissue for spatial omics sequencing (DBiT-seq), which employs microfluidic channels to print the array directly onto the tissue. Here, the spatial resolution depends on the diameter of the microfluidic channel used (no less than 10 μm) [115].

### 6.3. scRNA-seq Is a Key Tool for Deciphering the Complex Cellular Heterogeneity of the Cochlea

Previous studies on the auditory transcriptome were performed using microarray technologies and bulk RNA-seq [116], providing significant knowledge and information on the differential gene expression in physiological and pathological conditions of the cochlea, with important implications for the development of new therapies [117,118]. Studies on the transcriptome have also given insights into the developmental processes of the inner ear [119], the transcriptional changes associated with ageing [120], and cochlear cell damage/degeneration [121]. However, information on the specific cell populations is not possible with bulk RNA-seq, and this is a major limitation for studies on the cochlea due to its cellular heterogeneity [116].

In this context, scRNA-seq has given unprecedented information on cochlear cell diversity and alternative signaling mechanisms [122]. Profiling the transcriptome at a single-cell resolution helped to unravel novel populations of cells in the cochlea. For instance, subtypes of SCs (lateral and medial) with a distinct cluster of regenerative-associated markers were discovered in the avian cochlea [123]. Intriguingly, some of these discovered markers were also found to be expressed in specific regions of the mammalian cochlea [124], further supporting the stem cell-like potential of SCs [125]. Moreover, scRNA-seq has also allowed the identification of new specific markers of HCs, like sorcin (*sri*) for OHCs [126], which was then discovered to be implicated in calcium dynamics and the somatomotility of OHCs [126]. Another important application of scRNA-seq has been the study of exons and genes associated with deafness [126,127]. In this context, new genes associated with apoptosis, calcium regulation, and the extra cellular matrix (ECM) were found to be modulated in HCs of inner ear organoids in association with type II transmembrane protease 3 (tmprss3), a key gene for hearing loss [127]. Likewise, differential gene expression patterns among the cells of the lateral wall, the stria vascularis, and the immune system and SGNs have been identified in association with acoustic trauma by means of scRNA-seq, delineating a cell-specific transcriptomic map of the cochlea upon noise damage [128].

Overall, due to the complexity and heterogeneity of the cochlea, scRNA-seq has provided unmatched opportunities to broaden our current understanding of its molecular underpinnings in health and disease.

### 6.4. Spatial Transcriptomics Have Enabled Us to Understand the Cellular and Molecular Architecture of the Cochlea

The cochlea is spatially organized with distinct and localized functions. Thanks to the development of spatial transcriptomics methodologies, it is now possible to study the localization of specific gene expression patterns in relationship to the different anatomical structures of the cochlea [129,130]. For instance, the combination of scRNA-seq and FISH has allowed the identification of two subpopulations of SCs (named SC1 and SC2) retaining distinct transcriptomes in specific anatomic locations of the cochlea: medial for SC1 and lateral for SC2 [131]. Spatial transcriptomics are also particularly important for studies in the developing cochlea since the cochlea’s cellular organization during development is regulated by several spatiotemporal-dependent key signaling mechanisms. For instance, Munnamalai and co-workers investigated the spatiotemporal cadence of Wnt, NOTCH, and BMP signaling in the developing cochlea and found that they are differentially regulated depending on the cochlear location (from lateral to medial) and on the developmental stage. This study emphasizes the spatiotemporal signaling necessary to modulate the development of the cochlea in its radial axis and further supports the importance of spatial transcriptomics for cochlear research [132]. Another study used LCM-NGS to profile the transcriptome in different regions of the cochlea (e.g., the organ of Corti, spiral ganglion, lateral wall, and spiral limbus) and provided quantitative information on the transcripts of each region with important findings on deafness-associated genes [104].

To our knowledge, more advanced spatial transcriptomics technologies, like the Visium or the Stereo-seq technologies, have not yet been applied to cochlear research. However, it is expected that they could provide unparalleled opportunities for future studies in the field.

## 7. Epigenomics

### 7.1. Principles of Epigenomics

**Figure 4:**
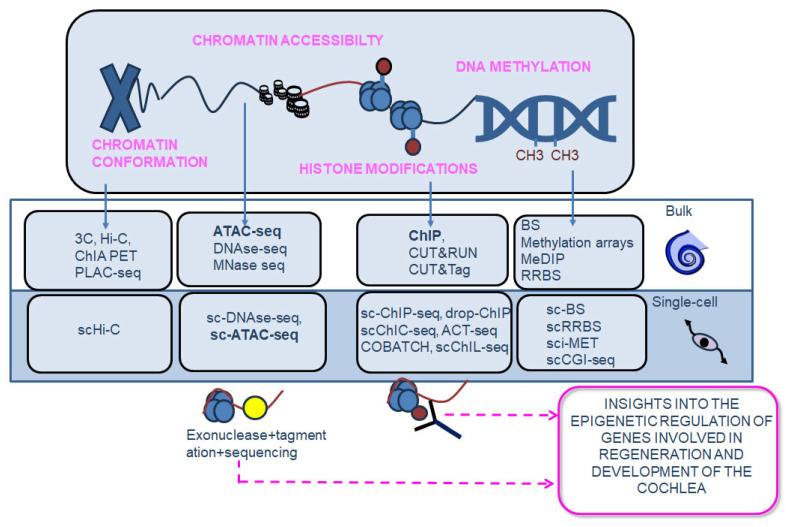
Schematic illustration of epigenomics. Epigenomics can be performed at a bulk, single, and spatial resolution. Bulk epigenomics and single-cell epigenomics have been applied in the cochlea. The study of epigenomics includes the assessment of DNA methylation dynamics, histone modifications, chromatin accessibility, and chromosome conformations. These epigenomic methods can be performed either with arrays or with sequencing. The techniques ChIP and ATAC-seq (in bulk and single cells) have been applied in the cochlea. ChIP relies on immunoprecipitating DNA–protein complexes via specific antibodies, and ATAC-seq uses Tn5 transposase chemistry and NGS to analyze open or accessible chromatin regions. These techniques have provided novel insights into the molecular mechanisms underlying the developmental and regenerative processes in the cochlea. *Abbreviations*: BS-seq, bisulfite sequencing; RRBS, reduced representation bisulfite sequencing; MeDIP, methylated DNA immunoprecipitation; ChIP, chromatin immunoprecipitation; CUT&RUN, cleavage under target and release using nuclease; CUT&Tag, ATAC-seq cleavage under targets and tagmentation; DNAse-seq, DNase I hyper-sensitive sites sequencing; FAIRE-seq, formaldehyde-assisted identification of regulatory elements followed by sequencing; 3C, conformation capture technique; PLAC-seq, proximity ligation-assisted ChIP-seq; ChIA PET, chromatin interaction analysis by paired-end tag sequencing; sci-MET, single-cell combinatorial indexing for methylation analysis; scCGI-seq, single-cell CGI methylation sequencing; scChIC-seq, single-cell chromatin immune-cleavage sequencing technique; ACT-seq, antibody-guided chromatin tagmentation sequencing; COBATCH, combinatorial barcoding and targeted chromatin release; scChIL-seq, single-cell chromatin integration labeling sequencing.

The term epigenomics refers to the techniques used to investigate the epigenome, which is the set of regulatory processes that modify the activity of gene expression without modifications in the DNA sequence. Epigenomics can be classified depending on the target: DNA methylation, histone modifications, chromatin accessibility, and chromosome interactions. The methodologies to study bulk epigenomics can be further classified as “array-based” and “sequencing-based” techniques [133]. Array-based technologies use hybridization with pre-designed microarrays, while sequencing-based methods use techniques like NGS. DNA methylation is an epigenetic mark where methyl groups are added to the cytosine bases of the DNA. It is important to highlight that to investigate DNA methylation, a required first step is the exposure of the methylated DNA through one of the following methods: (i) DNA digestion by methylation-sensitive restriction enzymes (MSREs) [134], (ii) affinity enrichment of DNA by antibodies targeting methylated CpGs [135], or (iii) conversion of unmethylated cytosines to uracil by bisulfite treatment [134]. To date, the bisulfite sequencing (BS-seq) method is considered the gold-standard technique for studies on DNA methylation because of its single-base resolution [133]. Histones can be modified primarily through acetylation, phosphorylation, methylation, and other miscellaneous modifications [136]. One of the most used techniques for monitoring histone modifications is chromatin immunoprecipitation (ChIP), in which the histone modifications of interest are targeted by antibodies. The cleavage under targets and release using nuclease (CUT&RUN) [137] and cleavage under targets and tagmentation (CUT&TAG) [138] methods are additional techniques used for the analysis of histone modifications; both rely on the same principle of recognizing DNA-bound proteins of interest through specific antibodies. The chromatin is highly dynamic, allowing regulators (enhancers, promoters, and chromatin-binding factors, among others) to have multiple physical interactions with DNA, thereby playing an important role in regulating gene expression. Multiple techniques for chromatin accessibility studies have also been developed. Among these, the most recent is the accessible chromatin using sequencing technology (ATAC-seq). It employs tagmentation (inserting adapter sequences by using the hyperactive mutant Tn5 transposase) to open target regions of the chromatin, which are then amplified and sequenced [139]. Other widely used techniques for chromatin accessibility include the DNase I hyper-sensitive sites sequencing (DNAse-seq) [140], the micrococcal nuclease digestion with deep sequencing (MNase-seq) [141], and the formaldehyde-assisted identification of regulatory elements followed by sequencing (FAIRE-seq) [142]. The higher-order organization of the nucleus is also important for the epigenetic regulation of cellular processes; hence, techniques able to analyze chromosomal interactions have also been developed. They include the chromatin conformation capture technique (3C), Hi-C, the chromatin interaction analysis by paired-end tag sequencing (ChIA PET), and the proximity ligation-assisted ChIP-seq (PLAC-seq) [133]. Further details of available epigenomics methodologies have already been extensively reviewed (see [133,143]), and those applied to cochlear research are summarized in Figure 4.

### 7.2. Single-Cell Epigenomics

Single-cell epigenomics enable a detailed analysis of the epigenetic regulation at a single-cell resolution, which includes single-cell DNA methylation profiling, single-cell chromatin mapping, single-cell Hi-C, and single-cell replication dynamics [144]. Several methods for single-cell DNA methylation profiling exist, the most recent of which are “single-cell combinatorial indexing for methylation analysis” (sci-MET) and “single-cell CGI methylation sequencing” (scCGI-seq) [133,145,146]. Histone modifications in single cells can also be studied using sc-ChIP-seq, single-cell droplet-based chromatin immunoprecipitation (drop-ChIP) [48], single-cell chromatin immune-cleavage sequencing technique (scChIC-seq), antibody-guided chromatin tagmentation sequencing (ACT-seq), combinatorial barcoding and targeted chromatin release (COBATCH), and single-cell chromatin integration labeling sequencing (scChIL-seq) [133]. Finally, single-cell chromatin accessibility can be investigated using scDNAse-seq and scATAC-seq. The available single-cell epigenetic methods have been recently reviewed in detail (see [144,147]).

### 7.3. Spatial Epigenomics

To fully appreciate the influence of epigenetic variations in patho-physiological processes, it is essential to know their spatial context. However, the development of spatial epigenomics techniques has been challenging for a long time due to the limited spatial resolution available [148,149]. The first spatial epigenomic technology was developed in 2021 and is now beginning to open new possibilities in the field of biology and medicine. The first spatial epigenomic technique that has been developed is the “high-spatial-resolution chromatin modification state profiling by sequencing” (hsrChST-seq). It is based on the spatial transcriptomic technique DBiT-seq, in which there is a combination of CUT&TAG and tissue deterministic barcoding with fluorescence microscopy [150]. Another technique developed later on to resolve chromatin accessibility spatially is the spatial-ATAC seq, which is based on the combination of in situ Tn5 transposase chemistry with microfluidic deterministic barcoding [151]. Recently, it has been possible to analyze the active and inactive promoters/enhancers associated with histone modifications in single cells while maintaining spatial information thanks to the advent of epigenomic MERFISH [151]. Epigenomic MERFISH combines CUT&TAG and MERFISH (a spatial epigenomic technique for the analysis of histone modifications) [152]. Furthermore, LCM can also be applied to epigenomics in order to spatially analyze modifications in the epigenome [153]. Finally, the most recent epigenomic technique is the spatial chromatin accessibility sequencing (SCA-seq), which provides simultaneous knowledge on the chromatin accessibility, epigenomics marks (e.g., CpG methylation), and higher-order genome architecture [154].

### 7.4. Epigenetic Profiling of the Cochlea Has Provided New Insights into the Mechanisms Whereby Genes Responsible for Auditory Function Are Regulated

Hearing loss can be caused by epigenetic alterations or by mutations in the genes encoding for the epigenetic machinery, affecting DNA methylation dynamics [155,156,157], histone modifications [158,159,160], and chromatin remodeling [156,161,162]. Thus, investigating epigenetic mechanisms could eventually pave the way towards new approaches to therapeutics. To date, most of the studies on the cochlear epigenome are based on bulk epigenomic profiling, and only a few were performed with single-cell epigenomics, namely scATAC-seq [163,164]. Instead, spatial epigenomics has not yet been applied in this field, though the epigenomics studies conducted until now have given us profound insights into the regulatory mechanisms of development, trans-differentiation, and regeneration of the auditory system. The application of ChIP-seq and ChIP-qPCR has led to the identification of fundamental epigenetic modifications in the promoters of two key genes involved in SGN differentiation (*Cdk2* and *NeuroD1*), which affects the binding of the regulatory transcription factor neurogenin 1 (neurog1) [165]. Also, ChIP-qPCR allowed for the description of the histone modifications associated with the epigenetic regulation of atonal bHLH transcription factor 1 (Atoh1), which is an evolutionarily conserved transcription factor for the development of the auditory system [166]. Yet, histone modifications of *Atoh1*, which are characteristic of HCs during their development, are suppressed in the same cells after birth, but they persist in perinatal SCs. This is an important finding, which gives new information on the mechanisms underlying the regenerative potential of SCs [166]. Likewise, the application of ATAC-seq provided new findings on specific variations in chromatin accessibility during the reprogramming of SCs into HCs in cochlear organoids [167], and scATAC-seq has unraveled important information on the mechanisms which limit the capacity of SCs trans-differentiation into HCs in the adult mammalian cochlea [163]. Furthermore, the combination of scATAC-seq with scRNA-seq has recently allowed the identification of molecular regulators of key transcription factors (such as Sox and Six) involved in HC regeneration from SCs in the zebrafish’s inner ear [164].

Overall, the epigenome profiling conducted until now in the cochlea has given new insights into the regulatory mechanisms of cochlear development, regeneration, and disease. It is expected that the application of the most advanced spatial epigenomics techniques in the future could provide a much better understanding of these processes.

## 8. Discussion

The cochlea is a complex sensory organ, whose degeneration can be caused by multiple damaging conditions that lead to irreversible hearing loss. The neuro-sensory epithelium—the organ of Corti—is particularly susceptible to degeneration as a consequence of inherited or environmental conditions. Moreover, SGNs degenerate as a consequence of damage to the organ of Corti, resulting in the reduced performance of cochlear implants [168,169,170]. As of now, there are no approved pharmacological treatments for SNHL. Recently, a novel putative therapeutic drug (FX-322) in a phase 2 clinical trial has been shown to hold promise in inducing the regeneration of HCs, thereby providing improvements in hearing function in cases of chronic noise-induced/sudden SNHL [171,172]. Hence, an understanding of the detailed molecular mechanisms that underlie cochlear physiology and pathology can pave the way for the development of new therapeutics to treat SNHL. The molecular basis (both genetic and non-genetic) of hearing loss, the developmental processes of the cochlea, and the stem cell-like regenerative potential of SCs in the organ of Corti are among the most active fields of cochlear research. Significant improvements in these areas have been possible due to the advent of advanced omics, and Table 2 summarizes such advanced genomics, transcriptomics, and epigenomics techniques that have been applied in cochlear research, alongside the improvement that they have provided in the field. For instance, genome sequencing has allowed for the identification of new variants in genetic hearing loss and represents a great improvement in the diagnosis of genetic deafness in newborns [82,83,84]. Nonetheless, studies on the cochlea are particularly challenging compared to other sensory organs because of several practical limitations. Indeed, the cochlea is enveloped in the bony labyrinth and cannot be visualized directly, requiring imaging techniques like MRI and CT [173,174] or histological techniques on postmortem tissues of humans and animals [175,176]. The high heterogeneity of the tissue [177], as well as the low number of cells, especially in the organ of Corti (~3.500 IHCs and ~12.000 OHCs in the human ear) [178], represent additional major limitations for studies in the field, especially with conventional molecular techniques (like real-time PCR or Western blotting). In general, there is a limited quantity of cells, nucleic acids, and other molecules (proteins and metabolites), which makes it necessary to pool samples in order to reach a sufficient amount for analysis. Moreover, the heterogeneity of the tissue in location (several subpopulations of cells with varied morphologies and functions) and direction (same population of cells varying in expression and phenotype) makes it difficult to interpret molecular data derived from a whole tissue. In fact, bulk studies in the whole cochlea may easily hide some molecular information, eventually diluted in the pool of the whole genome/transcriptome/epigenome. More recently, due to the advancements in single-cell omics techniques, these limitations have been successfully overcome [8]. In cochlear research, most of the single-cell studies have been performed to investigate the transcriptome through scRNA-seq [8,179]. Emerging single-cell transcriptomic techniques on live cells, such as live-seq, can be particularly useful in cochlear research in the future for two main reasons: (i) the live-seq technique is currently used for low sample input (as low as 1 pg) and (ii) the follow-up of a cell from its ground-state transcriptome to its downstream signaling pathways can enhance our understanding of processes of auditory function and development in health and disease. Single-cell analysis does not allow us to retain the spatial information, and the application of spatial transcriptomics seems to be of particular relevance in the field because of the complex cellular heterogeneity of the cochlea [129,132]. Until now, among spatial genomic, epigenomic, and transcriptomic methods, only the latter has been applied in cochlear research, to the best of our knowledge. Thanks to spatial transcriptomics, it has been possible to identify differential gene expression patterns for the development and regeneration of the cochlea in distinct cell populations of specific anatomical locations [180]. For instance, Waldhaus and colleagues profiled SCs in the apex and base of the murine cochlea and found that SCs—especially pillar cells—express regenerative and proliferative genes potentially relevant to the regeneration of HCs in mouse apical cochlea [180].

However, the most advanced spatial transcriptomic techniques, such as *visium* or stereo-seq, have not yet been exploited in cochlear research. Therefore, it is expected that using high-throughput resolution spatial omics will enable a further dissection of the cochlear patho-physiology in more detail. In addition to advanced single spatial omic techniques, spatial multi-omic methodologies, such as simultaneous profiling of the transcriptome and epigenome (both imaging-based and microfluidic deterministic barcoding-based methods), have emerged recently [181]. The application of such innovative techniques could provide an integrated picture of the multiple underlying molecular events, allowing a more comprehensive understanding of the cochlear function in health and disease. For instance, the genotype–phenotype correlation holds promise for the identification of novel biomarkers and pharmacological targets.

Nonetheless, thanks to the application of scRNA-seq in species capable of self-regenerating HCs (like zebrafish and birds), researchers have identified key expression patterns in SCs, which could potentially induce their reprogramming into HCs also in the mammalian cochlea [182,183]. Although some HC-like cells have been successfully regenerated in the murine cochlea by forcing the expression of the identified patterns, fully differentiated and functional HCs in the mammalian cochlea have not yet been developed. Notably, recent studies seem to indicate that this is due to epigenetic patterns. Indeed, epigenomic data obtained through scATAC-seq identified specific epigenetic modifications in the zebrafish’s SCs essential for their reprogramming into HCs [164]. Likewise, scATAC-seq also revealed the epigenetic mechanisms responsible for the inability of SCs to trans-differentiate into HCs in the mammalian cochlea [163]. From recent studies, the crucial role of epigenetic regulators in cochlear dynamics is apparent, but the sub-cellular/nuclear location of these epigenetic marks remains unknown. Hence, the application of spatial epigenomic techniques (such as epigenomic MERFISH) in addition to bulk/single-cell epigenomics can broaden our understanding of molecular regulation in auditory function. Although promising, epigenomic MERFISH is a targeted approach, and hence, prior knowledge on the epigenetic regulators is required. This strongly suggests a need for basic research in this area before the application of such high-throughput techniques.

The role of bioinformatics is undoubtedly crucial in maximizing the knowledge gained from omics data. Despite the huge amount of information provided by high-throughput omics methodologies, a major limitation lies in the storage of data and their interpretation. Indeed, multiple platforms are used for data storage, and each retains a different format; thus, the pre-processing of data is necessary before analysis. Moreover, artifacts may be generated for multiple reasons, such as the low amount of input material (especially for genome sequencing due to the presence of only two DNA copies) and the induction of stress genes due to the dissociation methods used for cell isolation. Therefore, orthogonal validation with targeted approaches is needed, and specialized bioinformaticians are necessary to properly read and interpret the data [47,88]. Although the currently available computational techniques have provided innumerable insights into the gained omics data in the cochlea, there are currently challenges encountered in efficient interpretation due to heterogeneous and noisy data, imbalance, or missing data, among others [51]. The application of the state-of-the-art deep learning models to analyzing spatial transcriptomics data such as spaCI [58] or SiGra [59] can indeed provide valuable information on cochlear cell architecture and dynamics.

**Table 2:** Advanced genomics, epigenomics, and transcriptomics techniques that have been applied in cochlear research.

Omics Categories	Techniques	Applications in Hearing Research	Models Utilized	Reference
Genomics	WGS, WES	Identification of novel structural variants and rare mutations in genes associated with deafness	Humans (affected individuals with the CRDHL)	[65]
	WES	Early detection of hearing loss for diagnostic purposes	Humans (individuals with diagnosis of hearing loss)	[83]
	Target exome panel	Improvement in the clinical diagnostic yield and thereby routine genetic screening	Humans (deaf patients suspected with underlying genetic causes of deafness)	[84]
			Humans (patients diagnosed with SNHL)	[82]
Transcriptomics	TruSeq	Identification of differential and preferential gene expression patterns and characterization of novel molecular pathways of the cochlea	Humans (patients with tumors of the skull base with normal hearing)	[118]
			Engineered mouse models of genes related to circadian rhythm with noise damage	[121]
		Comprehension of mechanisms involved in hair cell regeneration	Ototoxic (neomycin)-treated zebrafish	[184]
	SMART-Seq v4	Insights into the transcriptional changes in HCs during the process of ageing and damage	1-, 9-, 18-, 22-, and 26-month-old CBA/J mice	[120]
	RNA-Seq V2	Unraveling the genes specific to SGNs and their dynamicity in developmental processes	Mouse at different stages: E15.5, P1, P8, P14, and P30	[185]
Single-cell transcriptomics	SMART-Seq2	Identification of novel subtypes of cochlear cells	Chicken	[123]
		Identification of new markers of HCs	Mouse (C3HeB/FeJ)	[126]
	10x Genomics	Identification of gene regulatory networks involved in HC regeneration	Zebrafish (transgenic model for HC ablation)	[164]
		Identification of genes associated with *Tmprss3*-related hearing loss	Mouse (*Tmprss3-KO* organoids)	[127]
		Delineation of key regulatory mechanisms in HC regeneration	Rats	[186]
Spatial transcriptomics	Single-molecule FISH (smFISH)	Annotating distinct transcriptome of SC populations in specific anatomic locations of the cochlea	Mouse	[131]
	Whole-mount ISH	Spatiotemporal cadence of key signaling pathways in the context of developmental processes of the cochlea	Mouse organotypic cultures	[132]
			Genetically engineered mouse models of genes related to developmental processes	[129]
		Uncovering quantitative differential transcriptional profile in pre-mature and mature HCs, revealing novel role of genes in the differentiation process	Mice (P4 and 3 weeks old)	[130]
	LCM-NGS	Discovery of quantitative information of transcripts relevant in deafness in the organ of Corti, spiral ganglion, lateral wall, and spiral limbus	Mice (C57BL/6J)	[104]
Epigenomics	ChIP-seq and ChIP-qPCR	Epigenetic modifications in the promoters of genes involved in SGN differentiation	In vitro immortalized multipotent otic progenitors (iMOP cells)	[165]
	ATAC-seq	Identification of dynamics in chromatin accessibility of key transcriptional factors during the reprogramming of SCs into HCs	Mouse (Atoh1-nGFP, Sox2-GFP or Lgr5-GFP) and cochlear organoids	[167]
Single-cell epigenomics	scATAC-seq	Regulation of chromatin accessibility during the process of regeneration and identification of genetically conserved regenerative response elements necessary for injury/regenerative responses	Zebrafish (transgenic model for HC ablation)	[164]
		Identification of the epigenetic mechanisms responsible for the inability of SCs to trans-differentiate into HCs in the adult mammalian cochlea	Transgenic mouse models expressing transcription factors	[163]

## 9. Conclusions and Future Perspectives

Overall, advanced genomics, epigenomics, and transcriptomics techniques represent the state-of-the-art approaches in cochlear research and are providing unprecedented knowledge on the molecular basis of cochlear patho-physiology. There are various open questions in the field of cochlear research that could be addressed through the application of omic techniques. For instance, in the context of the heterogeneity of the cochlear tissue, as of now, eight different SC populations with distinct morphologies have been identified, but little is known about their contribution to auditory function [187]. The application of single-cell and spatial transcriptomic technologies could provide a map of cell-type-specific gene expression patterns that can help to hypothesize the potential role of these cells in auditory processes. In addition, the application of single-cell epigenomics could enhance our understanding of the important cell-type-specific gene regulatory networks that can broaden our knowledge on the putative role of SC in cochlear homeostasis. Another active area of research in hearing is the mechanisms underlying ototoxicity. For instance, cisplatin, one of the primary chemotherapeutic agents utilized in oncology, has debilitating effects on hearing function and, hence, quality of life. Although efforts have been undertaken to understand the molecular underpinnings behind cisplatin-induced ototoxicity, to date, no effective therapeutics have been approved to counteract such adverse effects [188]. This necessitates a thorough molecular comprehension of the ototoxic processes from cisplatin trafficking to downstream signaling. The application of single-cell and spatial omics can give insights into the consequences of those ototoxic compounds. For instance, genomics would provide advanced knowledge on the cell-type-specific mutations and their location, transcriptomics on the cell-type-specific biomarkers expressed under ototoxic conditions, and epigenomics on putative gene regulatory dynamics in ototoxicity, which can all provide novel pharmacological/gene therapy targets not only for HCs (where most of the research is focused on [189]) but also for SCs, which are known for their role in ototoxicity [190]. Furthermore, we do not yet fully understand the origin of various cell types present in the cochlea [191]. The application of single-cell omics can elucidate evident lineage-specific markers that can help trace back their origin. The identification of common/distinct progenitor/stem cell populations can not only provide clues on inner ear development but also can provide profound knowledge on cellular sources for regeneration and repair.

From the available literature, it is increasingly evident that the application of bulk/single-cell omics, as well as advanced multi-omics approaches, is necessary to achieve an integrated view of the biological processes of the cochlea in health, development, and disease. Further studies combining genomics/transcriptomics/epigenomics with other omics—like proteomics and metabolomics—will give unmatched opportunities to decipher the complex molecular underpinnings of such a complex sensory organ.

## Figures and Tables

**Table 1:** Advanced sequencing methodologies for nucleic acids.

Sequencing Technology	Category	Principle	Read Length	Reference
NGS	Cyclic-array sequencing (Illumina and Ion Torrent)	Repeated cycles of enzymatic catalytic reactions.	Short	[67]
NGS	Hybridization-based sequencing	Multiple oligonucleotides are hybridized with complementary sequences of the target genome/transcriptome.	Short	[68]
NGS	Microelectrophoretic-based	Lab-on-a-chip level which combines all the Sanger sequencing steps together for a more efficient sequencing.	Short	[69]
TGS	Pacific Biosciences (PacBio)	Laser-induced fluorescence signals that are activated during the incorporation of dNTPs into DNA, alongside recording the color and duration of the signals in real time.	Long	[70]
TGS	Oxford nanopore technology (ONT)	Nanopore-based technology in which sequencing is allowed by determination of current change induced by nucleotides passing through the nanopore.	Long	[71]

## Data Availability

Data sharing not applicable.
